# Overexpression of phosphatidylserine synthase *IbPSS1* affords cellular Na^+^ homeostasis and salt tolerance by activating plasma membrane Na^+^/H^+^ antiport activity in sweet potato roots

**DOI:** 10.1038/s41438-020-00358-1

**Published:** 2020-08-01

**Authors:** Yicheng Yu, Ying Xuan, Xiaofeng Bian, Lei Zhang, Zhiyuan Pan, Meng Kou, Qinghe Cao, Zhonghou Tang, Qiang Li, Daifu Ma, Zongyun Li, Jian Sun

**Affiliations:** 1 Jiangsu Key Laboratory of Phylogenomics and Comparative Genomics, School of Life Sciences, Jiangsu Normal University, 221116 Xuzhou, Jiangsu China; 2 Institute of Food Crops, Provincial Key Laboratory of Agrobiology, Jiangsu Academy of Agricultural Sciences, 210014 Nanjing, China; 3 Xuzhou Institute of Agricultural Sciences in Jiangsu Xuhuai District, 221131 Xuzhou, Jiangsu Province China

**Keywords:** Transgenic plants, Salt

## Abstract

Phosphatidylserine synthase (PSS)-mediated phosphatidylserine (PS) synthesis is crucial for plant development. However, little is known about the contribution of PSS to Na^+^ homeostasis regulation and salt tolerance in plants. Here, we cloned the *IbPSS1* gene, which encodes an ortholog of *Arabidopsis AtPSS1*, from sweet potato (*Ipomoea batatas* (L.) Lam.). The transient expression of *IbPSS1* in *Nicotiana benthamiana* leaves increased PS abundance. We then established an efficient *Agrobacterium rhizogenes*-mediated in vivo root transgenic system for sweet potato. Overexpression of *IbPSS1* through this system markedly decreased cellular Na^+^ accumulation in salinized transgenic roots (TRs) compared with adventitious roots. The overexpression of *IbPSS1* enhanced salt-induced Na^+^/H^+^ antiport activity and increased plasma membrane (PM) Ca^2+^-permeable channel sensitivity to NaCl and H_2_O_2_ in the TRs. We confirmed the important role of *IbPSS1* in improving salt tolerance in transgenic sweet potato lines obtained from an *Agrobacterium tumefaciens*-mediated transformation system. Similarly, compared with the wild-type (WT) plants, the transgenic lines presented decreased Na^+^ accumulation, enhanced Na^+^ exclusion, and increased PM Ca^2+^-permeable channel sensitivity to NaCl and H_2_O_2_ in the roots. Exogenous application of lysophosphatidylserine triggered similar shifts in Na^+^ accumulation and Na^+^ and Ca^2+^ fluxes in the salinized roots of WT. Overall, this study provides an efficient and reliable transgenic method for functional genomic studies of sweet potato. Our results revealed that *IbPSS1* contributes to the salt tolerance of sweet potato by enabling Na^+^ homeostasis and Na^+^ exclusion in the roots, and the latter process is possibly controlled by PS reinforcing Ca^2+^ signaling in the roots.

## Introduction

High concentrations of NaCl in the soil disrupt plant growth, cellular K^+^/Na^+^ homeostasis, and metabolic processes and markedly decrease crop yield in irrigated lands^1^. The increase in Na^+^ extrusion from the roots, reduction in root-to-shoot Na^+^ translocation, compartmentalization of Na^+^ in the vacuole, and maintenance of appropriate cytoplasmic K^+^ abundance are essential for plant salt tolerance^1^. For example, many plants activate plasma membrane (PM) Na^+^/H^+^ antiporter-mediated Na^+^ exclusion to maintain Na^+^ homeostasis in the cytosol under saline conditions^1,2^. Furthermore, the conserved salt overly sensitive (SOS) pathway regulates PM Na^+^/H^+^ antiporter activation in plants; in this pathway, a salt-induced buildup of cytosolic Ca^2+^ ([Ca^2+^]_cyt_) is identified by SOS3/CBL4 calcium sensors, which bind to SOS2/CIPK24 to form a complex^2^. The SOS2–SOS3 complex ultimately phosphorylates SOS1/NHX7 (a PM Na^+^/H^+^ antiporter), which can export Na^+^ from the cell^2^. Salt stress rapidly triggers an apoplastic H_2_O_2_ burst, which promotes the mediation of Na^+^ homeostasis in various plant species^3–5^. Chemical inhibition or mutations of PM NADPH oxidase decrease salt tolerance by reducing the salt-induced Ca^2+^ influx across the PM and the subsequent increase in [Ca^2+^]_cyt_^3,6^. These findings suggest that H_2_O_2_ activates PM Ca^2+^-permeable channels to enhance the signaling of [Ca^2+^]_cyt_ under saline conditions^3,6^.

Lipid remodeling plays a critical role in plant salt tolerance because lipids affect membrane integrity, fluidity and membrane protein activity and function^7^. Alterations in membrane lipid composition act as signaling agents that mediate salt defensive responses in plants^1^. Phosphatidic acid (PA), phosphoinositide derivatives, and sphingolipids are important regulators of [Ca^2+^]_cyt_ signaling, ion homeostasis, and salt tolerance in plants^8,9^. Phosphoinositide-phospholipase C (OsPLC1) is required for the hydrolyzation of phosphatidylinositol-4-phosphate and the formation of a salt-induced [Ca^2+^]_cyt_ signature, which determines Na^+^ accumulation in leaf blades and whole-plant tolerance in rice^10^. PA production is induced rapidly in response to salt stress and plays multiple roles in mediating salt tolerance, such as the regulation of auxin efflux transporters^11^, mediation of microtubule organization^12^, stimulation of MPK6 activity to activate Na^+^/H^+^ exchange across the PM, and coordination of cellular ROS and pH signaling^13–15^. Recently, glycosyl inositol phosphorylceramide (GIPC) sphingolipids in the PM have been identified as a cell surface Na^+^ receptor required for NaCl-induced [Ca^2+^]_cyt_ increase, activation of PM Na^+^/H^+^ antiporters, and basal salt tolerance in the model plant species *Arabidopsis thaliana*^16^.

Phosphatidylserine (PS) is a negatively charged phospholipid that is synthesized in the luminal leaflet of the endoplasmic reticulum by phosphatidylserine synthase (PSS); it must be flipped into the inner leaflet of the PM to contribute to membrane structure and electrostatic interactions in animals and plants^17^. Similar to other anionic phospholipids, the PS in the PM contributes to electrostatic interactions with other functional proteins and plays essential regulatory roles in many biological processes in animals and plants^17^. In *Drosophila*, PSS-mediated PS production mediates cell growth, lipid storage, and mitochondrial function^18^. In *A. thaliana*, AtPSS1, which catalyzes PS production by calcium-dependent base-exchange-type reactions with other phospholipids, is required for the development of microspores, inflorescence meristems, leaves, and internodes^19,20^. In rice (*Oryza sativa* L.) plants, PSS is involved in the elongation of cells in the uppermost internode and leaf senescence regulation^21–23^. Experimental manipulation of PS abundance in the PM by knocking down or overexpressing the Arabidopsis *AtPSS1* gene leads to the graded activation of the PM auxin signaling pathway, which is regulated by the small GTPase ROP6^24^. Moreover, PS is essential for the proper targeting of ROP6 to the PM, suggesting the essential signaling roles of PS in the mediation of plant development^24^. Our previous work revealed that salt-induced increases in PS facilitate ion homeostasis and tissue tolerance to salt stress in sweet potato^25^, suggesting that PSS is an important candidate gene that can be used to improve crop salt tolerance. However, whether PSS regulates salt defensive responses and salt tolerance in plants remains to be elucidated.

Herein, we demonstrated that the overexpression of a sweet potato PSS, *IbPSS1*, which is an ortholog of Arabidopsis *AtPSS1*, promotes cellular Na^+^ homeostasis by activating PM Na^+^/H^+^ antiport activity in sweet potato roots. Moreover, we showed that *IbPSS1* overexpression enhances PM Ca^2+^-permeable channel sensitivity to NaCl and H_2_O_2_. Taken together, the results show that *IbPSS1* acts as a key regulator of Na^+^ homeostasis, Ca^2+^ signaling, and salt tolerance in sweet potato.

## Results

### Identification of IbPSS1 from sweet potato

**Fig. 1:**
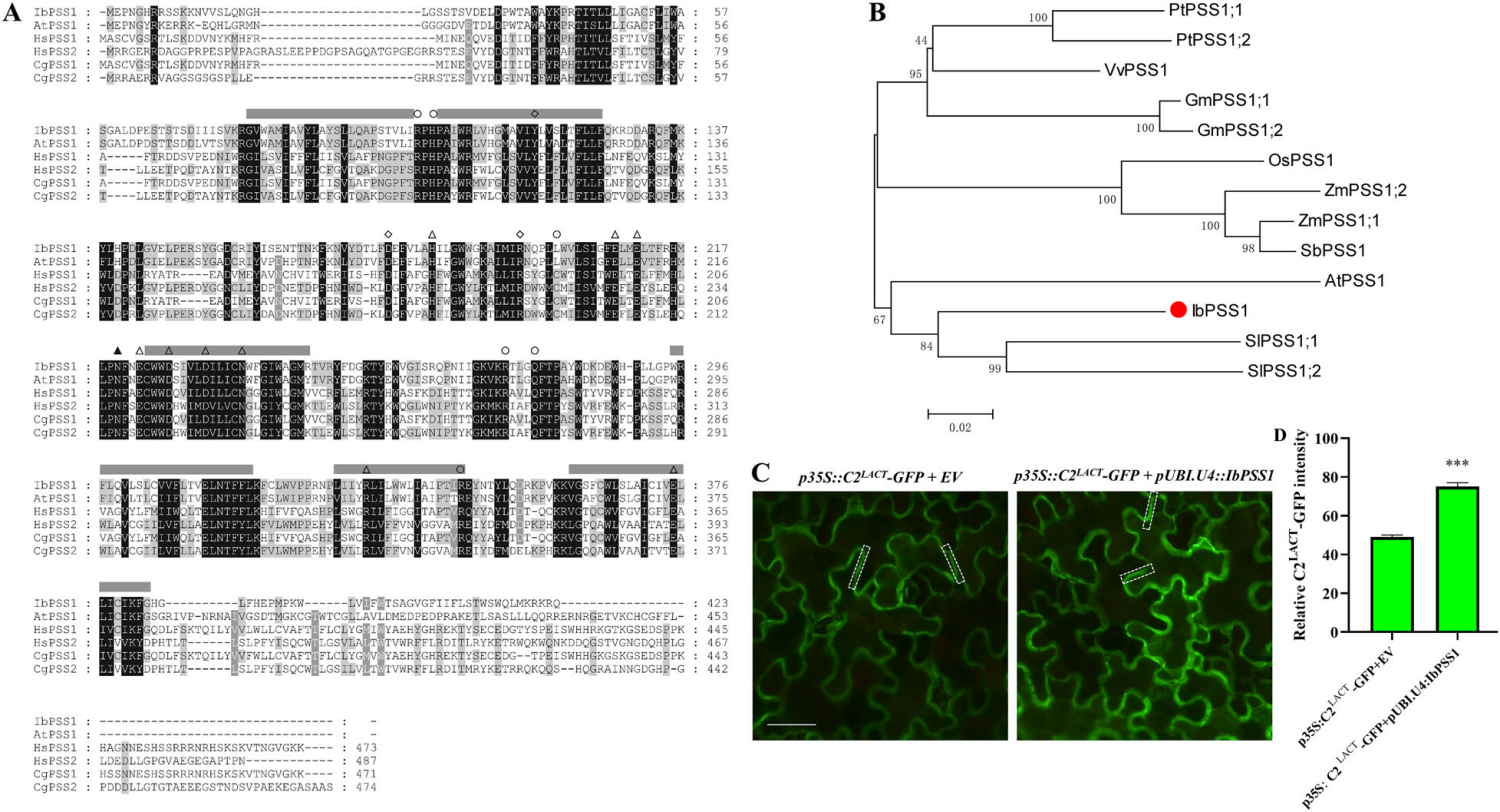
IbPSS1 is a base-exchange-type phosphatidylserine synthase (PSS) that contributes to PS synthesis. **a** Multiple alignment of base-exchange-type PSS protein sequences from *Homo sapiens* (*Hs*), *Cricetulus griseus* (*Cg*), *Ipomoea batatas* (*Ib*) and *Arabidopsis thaliana* (*At*). The amino acid residues indicated are important for CgPSS1 catalytic activity (open triangles), free Ser binding/recognition (closed triangle), PSS1 regulation (open circles), and PSS production and/or stability (diamonds). The black boxes indicate conserved residues. The gray bars indicate hypothetical membrane-spanning domains. **b** Phylogenetic analysis of IbPSS1 and its orthologs from different plant species. PSS1 proteins from the different species are indicated as follows: AtPSS1, *Arabidopsis thaliana*; GmPSS1;1 and GmPSS1;2, *Glycine max*; OsPSS1, *Oryza sativa*; PtPSS1;1 and PtPSS1;2, *Populus trichocarpa*; SbPSS1, *Sorghum bicolor*; SlPSS1;1 and SlPSS1;2, *Solanum lycopersicum*; VvPSS1, *Vitis vinifera*; and ZmPSS1;1 and ZmPSS1;2, *Zea mays*. The numbers on the tree indicate bootstrap supports (values < 50% are not shown). The branch lengths were drawn to scale; the bar size indicates the number of amino acid substitutions per site. **c** The transient expression of *IbPSS1* in *Nicotiana benthamiana* leaves increases PS abundance in the PM region. A typical image of one of three independent experiments is shown. Bar = 30 μm. **e** Quantification of C2^LACT^-GFP fluorescence intensity. The columns labeled with “***” indicate significant differences at *P* < 0.001. *n* > 100 PM regions from at least 40 cells in three independent experiments.

A PSS gene was isolated from the sweet potato cultivar Xushu 29 (Xu 29). The ORF of this gene is 1272 bp in length and encodes a 423 aa polypeptide (molecular weight: 49.4 kDa) with a predicted pI of 9.12. Multiple sequence alignment of this PSS and its orthologs from different species showed that it is a calcium-dependent base-exchange-type PSS (Fig. 1a). Phylogenetic analysis of the orthologs of PSS from different plant species indicated that this PSS gene from sweet potato is closely related to its orthologs in tomato (*SlPSS1;1* and *SlPSS1;2*) and Arabidopsis (*AtPSS1*) (Fig. 1b). Thus, we named this gene *IbPSS1* (GenBank accession number MN857546).

To confirm whether *IbPSS1* contributes to PS synthesis, we transiently coexpressed *IbPSS1* with a genetically encoded biosensor of PS (C2^LACT^-GFP) in *Nicotiana benthamiana* leaves. This biosensor was extensively validated as a calcium-independent-specific PS reporter^17,24^. Figure 1c shows that the green fluorescence of C2^LACT^-GFP was mainly distributed in the marginal region of epidermal cells with or without *IbPSS1* expression, suggesting that PS is mainly localized in the PM. However, the relative fluorescence intensity in the PM region of *IbPSS1* and C2^LACT^-GFP coexpressed cells was significantly higher than that of C2^LACT^-GFP and empty vector (EV) coinoculated cells (Fig. 1d). These results confirmed that *IbPSS1* contributes to the synthesis of PS.

### Establishment of an efficient root transgenic system in sweet potato

**Fig. 2:**
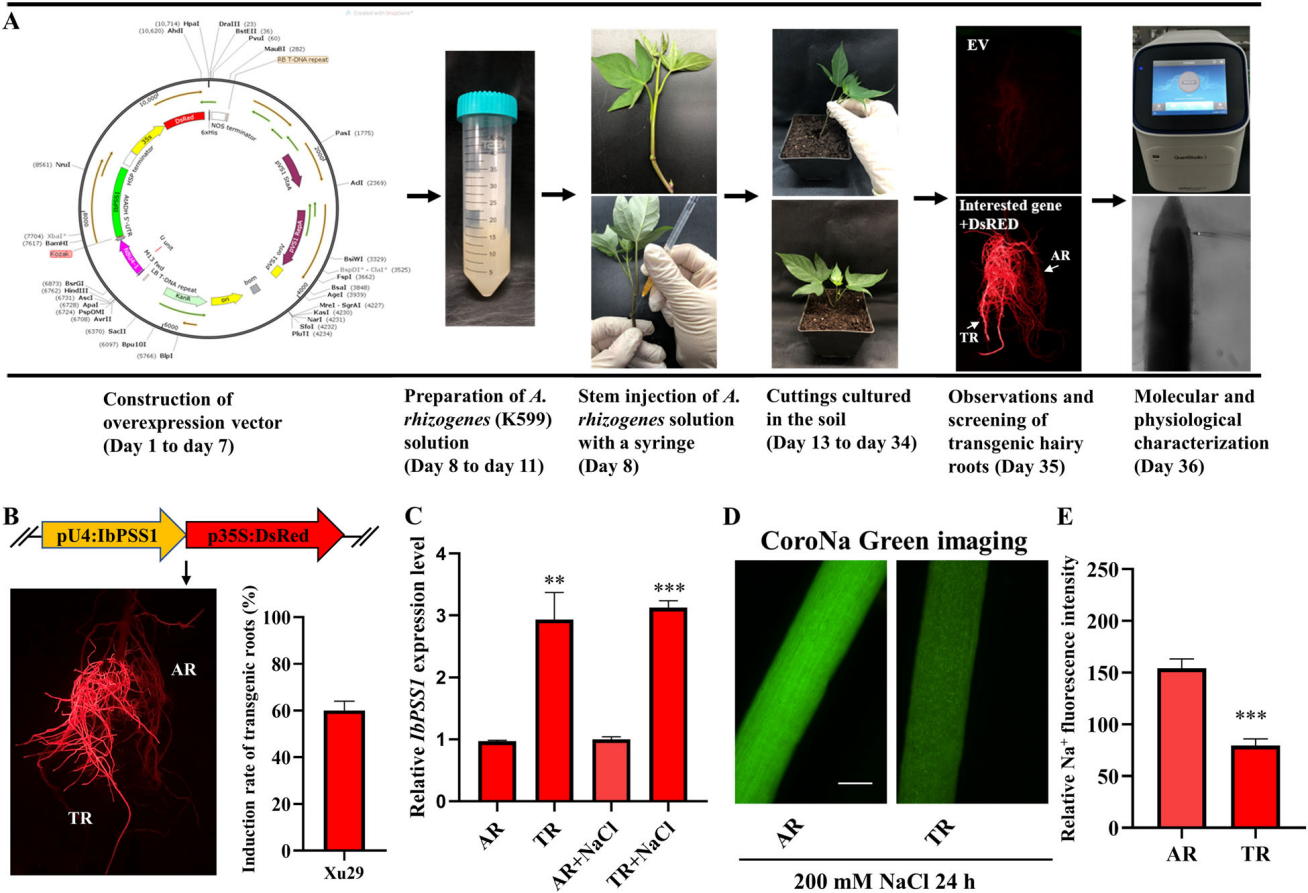
Overexpression of *IbPSS1* using the root transgenic system decreases Na^+^ accumulation in salinized sweet potato (Xu 29) roots. **a** Pipeline of the *A. rhizogenes*-mediated in vivo root transgenic system. TRs: transgenic roots; ARs: adventitious roots. **b** Schematic of *pUBI.U4::IbPSS1-CaMV35S::DsRed* expression cassette, representative images of the TRs and ARs in the same seedling and the induction rate of TRs for this construct. **c** Relative expression level of *IbPSS1* in the TRs and ARs with or without NaCl treatment (200 mM for 24 h). **d** Na^+^ accumulation in the elongation zones of the TRs and ARs. A typical image (*n* = 15 from three experiments) is shown. Bar = 0.3 mm. **e** Quantification of Na^+^ fluorescence intensity. The columns labeled with “***” indicate significant differences at *P* < 0.001.

To rapidly determine the role of *IbPSS1* in the mediation of Na^+^ homeostasis, we established an efficient *A. rhizogenes*-mediated in vivo root transgenic system in sweet potato^26^ (Fig. 2a). In our system, we introduced the expression vector containing two expression cassettes (the gene of interest + the *DsRed* marker) into *A. rhizogenes* strain K599, which was then injected into the basal part of stems of uniform cuttings of sweet potato cultivars. After growing in soil for 3 weeks, the rooted sweet potato seedlings were removed and washed, and many roots with obvious red fluorescence were visible around the injection site (Fig. 2a). The *A. rhizogenes*-mediated induction of transgenic roots (TRs) exhibited high efficiency in some sweet potato cultivars (*DsRed* only). For example, a 95% induction rate and an 80% induction rate were observed in the purple sweet potato Xuzishu 8 (Zi 8) and the yellow-flesh sweet potato Xu 29, respectively (Fig. S1).

To verify whether the root transgenic system reflects the influence of the genes of interest on Na^+^ homeostasis in root cells, we selected *IbSOS1*, which encodes a PM Na^+^/H^+^ antiporter in sweet potato, to confirm the effectiveness of the experimental system. We constructed a vector harboring two expression cassettes—one for *IbSOS1* and one for the reporter gene *DsRed*—and then transferred the vector into Zi 8 as described above. After 3 weeks, many TRs with bright red fluorescence were obtained (Fig. S2A). The average transformation rate reached 85% for this vector (Fig. S2B). qRT-PCR data confirmed that *IbSOS1* was successfully overexpressed in the TRs (Fig. S2C). We used CoroNa™ Green-based Na^+^ imaging and noninvasive microelectrode Na^+^ flux measurements to estimate the influence of *IbSOS1* on root Na^+^ homeostasis in sweet potato^5,27–29^. After 24 h of NaCl stress (200 mM), bright CoroNa™ Green-specific fluorescence was recorded in the elongation zone in most adventitious roots (ARs) (Fig. S2D). However, Na^+^ accumulation in the elongation zone was markedly lower in the TRs than in the ARs (Fig. S2D, E). In addition, the Na^+^ efflux recorded in the elongation and mature zones of the TRs was significantly higher than that of the ARs (Figs S2F and G). Moreover, the Na^+^ efflux did not differ between the *DsRed* TRs (positive control) and the ARs under saline conditions (Fig. S3). These results clearly showed that our system can efficiently be used to determine the role of genes of interest in mediating Na^+^ homeostasis in sweet potato roots.

### Cellular Na^+^ homeostasis is altered in IbPSS1-overexpressing sweet potato roots

We further tested whether *IbPSS1* influences root Na^+^ homeostasis by using this root transgenic system. We constructed a vector with two expression cassettes—one for *IbPSS1* and one for *DsRed*—and then transferred the vector into Xu 29. The average induction rate of the TRs reached 60% for this vector in four independent experiments (Fig. 2b). The relative expression level of *IbPSS1* in the TRs was higher (3-fold) than that in the ARs under both control and saline conditions (Fig. 2c), suggesting that *IbPSS1* was successfully overexpressed in sweet potato roots. However, the *IbPSS1* transcript level did not increase in the ARs or TRs in response to NaCl treatment (Fig. 2c), suggesting that this gene was not upregulated by NaCl in the roots of this cultivar. After 24 h of NaCl stress (200 mM), we observed an obviously high accumulation of Na^+^, as indicated by CoroNa^TM^ Green fluorescence, in the elongation zone of the ARs compared with the TRs (Fig. 2d). In addition, the relative Na^+^ fluorescence was reduced by 51% in the *IbPSS1*-overexpressing roots compared with the ARs (Fig. 2e). These results suggest that *IbPSS1* overexpression contributes to the inhibition of Na^+^ accumulation in salinized sweet potato roots.

**Fig. 3:**
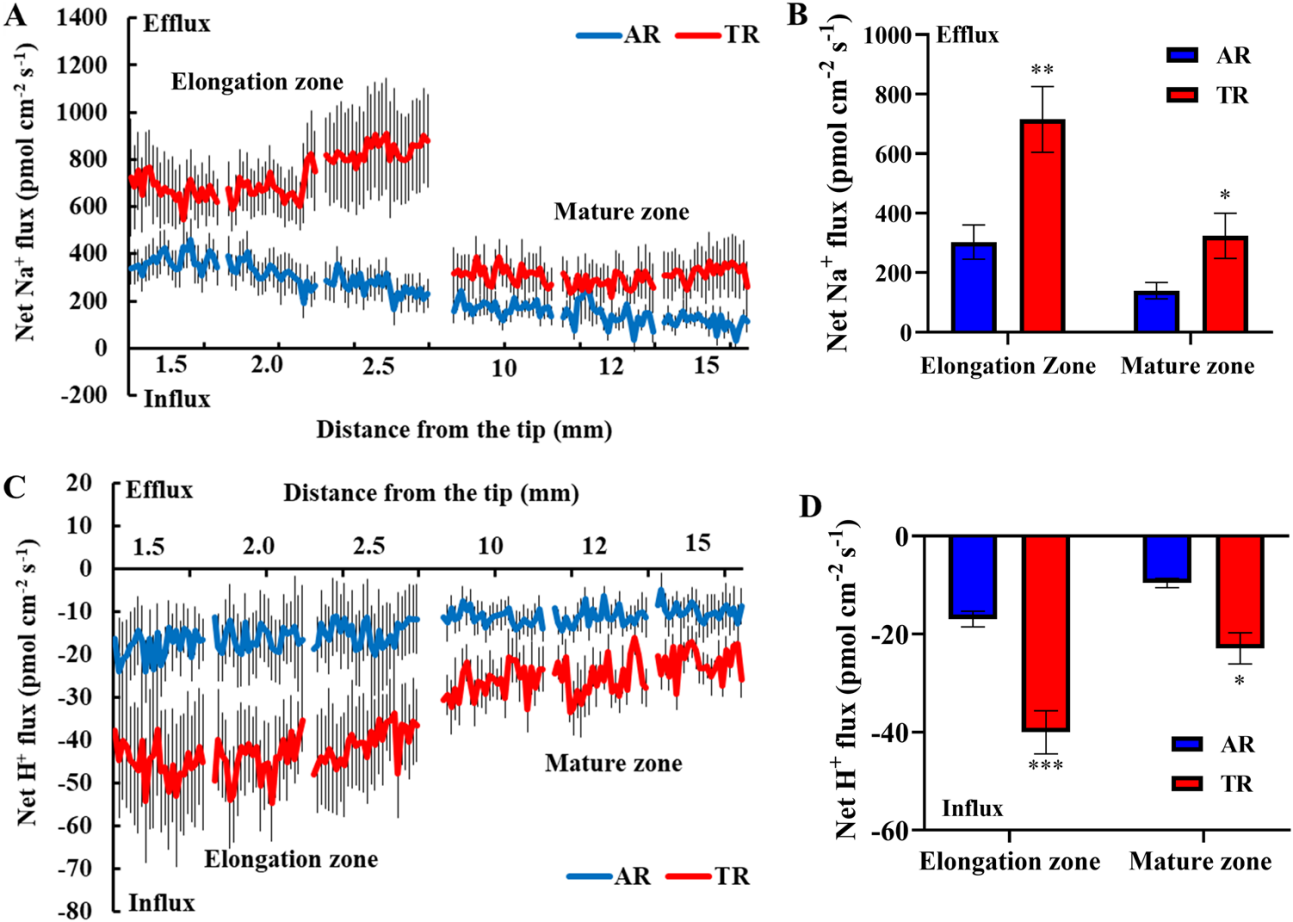
Overexpression of *IbPSS1* in sweet potato roots enhances NaCl-induced Na^+^/H^+^ antiport activity. **a**, **c** Net Na^+^ and H^+^ fluxes in the ARs and TRs measured by NMT. The steady-state Na^+^ and H^+^ fluxes were measured from the elongation (1.5, 2.0, and 3.0 mm from the tip) and mature root zones (10, 12, and 15 mm from the tip) after 24 h of NaCl treatment (200 mM). *n* > 20 from ten individual seedlings. The standard errors of the means are represented by the bars. **b**, **d** Mean net Na^+^ efflux and net H^+^ influx rates in (**a**) and (**b**), respectively. The columns labeled with “**” and “***” indicate significant differences between the AR and TR groups at *P* < 0.01 and *P* < 0.001, respectively.

To explore how *IbPSS1* inhibits root Na^+^ accumulation, we measured the salt-induced Na^+^ and H^+^ flux, which reflects the PM Na^+^/H^+^ exchange activity, in various plant species^27,29^ from different zones of the ARs and TRs by using noninvasive microtest technology (NMT). After 24 h of NaCl stress (200 mM), all tested root zones (elongation and mature zones) of the ARs and TRs showed evident Na^+^ efflux (Fig. 3a), which indicates that NaCl activated Na^+^ extrusion activity. Interestingly, a more pronounced Na^+^ efflux was observed in the TRs than in the ARs. The mean Na^+^ efflux rates in the two root zones of the TRs were 2.5- and 2.3-fold higher than those of the ARs, respectively (Fig. 3a, b). Correspondingly, evident H^+^ influx was recorded in the two root zones of the ARs and TRs (Fig. 3c), mirroring the Na^+^/H^+^ antiporter activity across the PMs of the root cells. Similar to the Na^+^ extrusion activity, the H^+^ influx was markedly higher in the two root zones of the TRs than in those of the ARs (Fig. 3d). No difference in the Na^+^ or H^+^ flux was observed between the ARs and TRs without NaCl stress (data not shown). These results indicate that *IbPSS1* overexpression inhibits cellular Na^+^ accumulation by activating PM Na^+^/H^+^ antiporter activity in sweet potato roots. Interestingly, no significant difference in *IbSOS1* transcript levels were found between the ARs and TRs under the control and saline conditions (Fig. S4). Moreover, *IbPSS1* overexpression in sweet potato roots also inhibited K^+^ efflux induced by salt in the root elongation zone (Fig. S5).

### IbPSS1 overexpression in sweet potato roots enhances PM Ca^2+^-permeable channel sensitivity to NaCl and H_2_O_2_

**Fig. 4:**
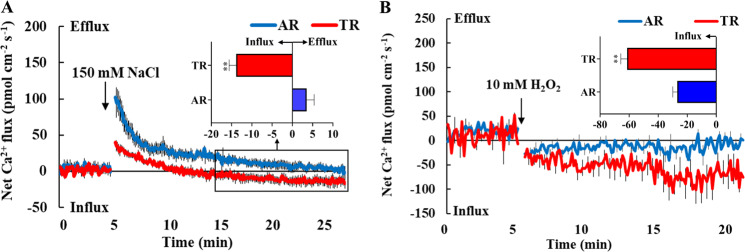
Overexpression of *IbPSS1* in sweet potato roots alters Ca^2+^ flux kinetics in response to NaCl and H_2_O_2_ stimuli. **a** Effects of NaCl stress (150 mM) on transient net Ca^2+^ flux kinetics in the elongation zone (2 mm from the tip) of the TRs and ARs. *n* = 9 from five individual seedlings. The columns in the insert show the mean rates of Ca^2+^ flux during the later period of NaCl stress (~10 min). “**” denotes significant differences at *P* < *0.01*. **b** Effects of H_2_O_2_ (10 mM) on transient net Ca^2+^ flux kinetics in the elongation zones (2 mm from the tip) of the TRs and ARs. *n* = 12 from six individual seedlings. The columns in the insert show the mean Ca^2+^ flux rates during the H_2_O_2_ treatment (~20 min). “**” denotes significant differences at *P* < 0.01.

The salt-triggered Na^+^ extrusion to the apoplast in plant roots is generally accompanied by a Ca^2+^ influx across the PM, which contributes to the [Ca^2+^]_cyt_ increase and PM Na^+^/H^+^ antiporter activation via the SOS pathway^3,5,16^. To examine whether *IbPSS1* influences Ca^2+^ transport in salinized sweet potato roots, we determined the NaCl-induced transient Ca^2+^ flux kinetics. NaCl (150 mM) triggered an instantaneous and gradually decreasing Ca^2+^ efflux in the elongation zone of the ARs. This salt-induced Ca^2+^ efflux recovered to the control level after 20 min of NaCl treatment (Fig. 4a). We did not observe any evident increase in Ca^2+^ influx during salt stress in the ARs. However, NaCl induced a much lower Ca^2+^ efflux in the elongation zone of the TRs than in that of the ARs (Fig. 4a). In TRs, we observed a gradual decrease in NaCl-triggered Ca^2+^ efflux from 0 to 10 min after the addition of NaCl and evident transition of Ca^2+^ efflux to Ca^2+^ influx after 10 min of NaCl treatment (Fig. 4a). These results clearly show that *IbPSS1* overexpression enhances PM Ca^2+^-permeable channel sensitivity to NaCl stress in sweet potato roots.

H_2_O_2_-activated PM Ca^2+^ channels contribute to PM Na^+^/H^+^ antiporter activation under saline conditions^3,5^. We further compared the Ca^2+^ flux kinetics induced by H_2_O_2_ in the elongation zone of ARs and TRs. H_2_O_2_ (10 mM) triggered an immediate Ca^2+^ influx in the ARs. The mean Ca^2+^ influx rate under H_2_O_2_ treatment reached 23 pmol cm^−2^ s^−1^ (Fig. 4b). However, H_2_O_2_ induced a more pronounced Ca^2+^ influx in the elongation zone of the TRs than in that of the ARs. The mean Ca^2+^ influx rate in the TRs reached 60 pmol cm^−2^ s^−1^ (Fig. 4b). Together, these results indicate that the PM Ca^2+^-permeable channel sensitivity to H_2_O_2_ is also reinforced in the TRs.

### IbPSS1 overexpression enhances salt tolerance in transgenic sweet potato at the whole-plant level

**Fig. 5:**
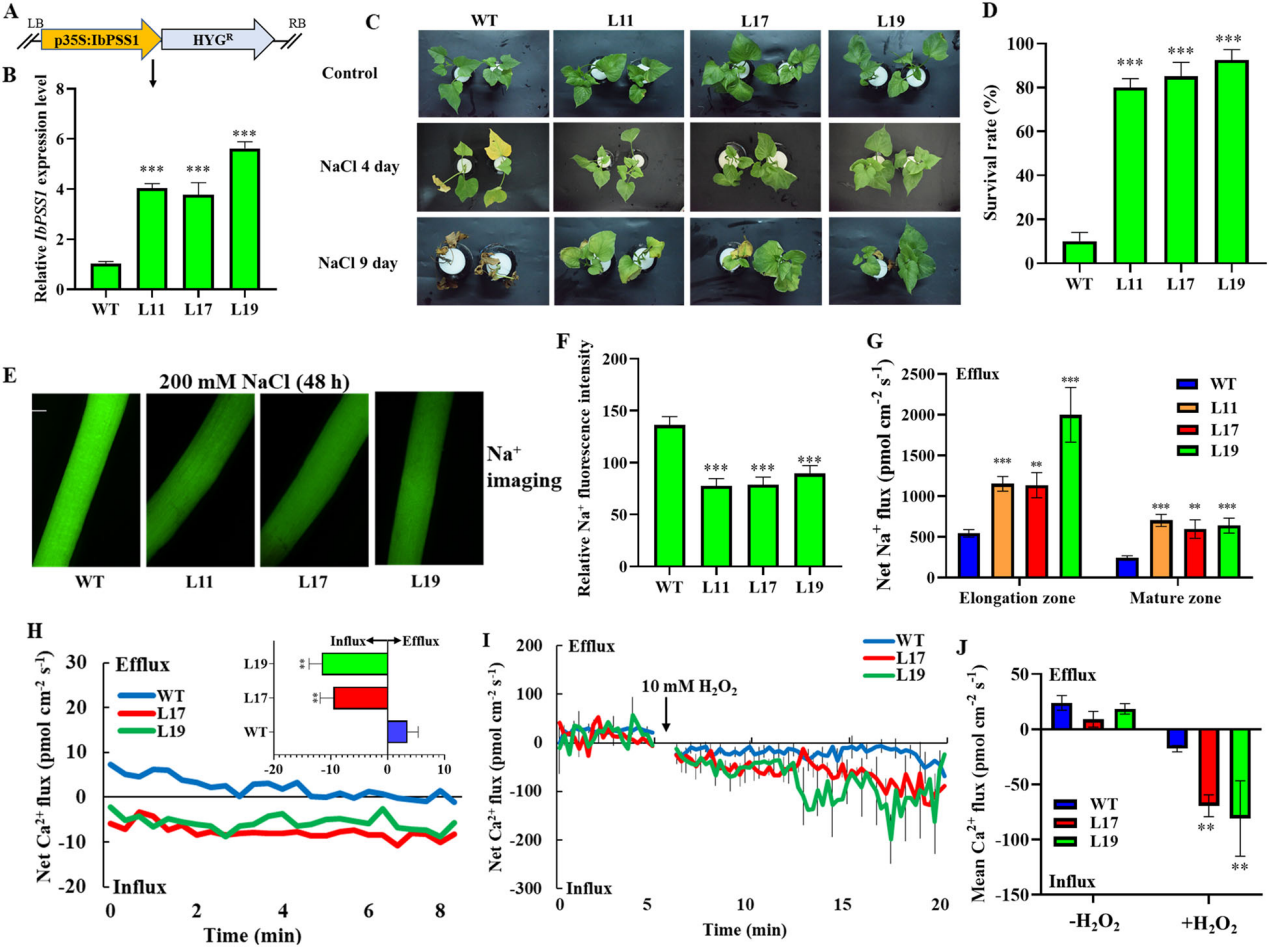
Overexpression of *IbPSS1* in sweet potato (Xu 29) enhances salt tolerance at the whole-plant level by reinforcing Na^+^ extrusion and Ca^2+^ influx in the roots. **a** Schematic of the *pCaMV35S::IbPSS1* expression cassette. **b** Relative expression level of *IbPSS1* in the wild type (WT) and three independent transgenic lines. **c** Representative growth phenotype of WT and transgenic lines under control and saline conditions (200 mM NaCl for 4 and 9 days). **d** Quantification of the survival rate from four independent experiments. **e** Na^+^ accumulation in the root elongation zones of the WT and transgenic lines. A typical image (of 20) is shown. Bar = 0.3 mm. **f** Quantification of Na^+^ fluorescence intensity. **g** Net Na^+^ flux measured by NMT in the elongation and mature root zones of the WT and transgenic lines. *n* = 12 from six individual seedlings. The standard errors of the means are represented by the bars. **h** Effects of NaCl stress (150 mM) on the Ca^2+^ flux in the root elongation zone (2 mm from the tip) in the WT and transgenic lines. Ca^2+^ flux measurements were started after 10 min of NaCl stress. The inserted column shows the mean Ca^2+^ flux rate during the measurement period. *n* = 8 from four individual seedlings. **i** Effects of H_2_O_2_ (10 mM) on the transient net Ca^2+^ flux kinetics in the root elongation zones (2 mm from the tip) of the WT and transgenic lines. *n* = 8 from four individual seedlings. **j** Histogram showing the mean rate of Ca^2+^ flux before (~5 min) and after (15 min) the addition of H_2_O_2_. For all the histograms, the columns labeled with “*,” “**,” and “***” indicate significant differences at *P* < 0.05, *P* < 0.01, and *P* < 0.001, respectively, compared with the WT.

To confirm whether *IbPSS1* could enhance salt tolerance at the whole-plant level, *A. tumefaciens*-mediated transformation was used to introduce the *pCaMV35S:IbPSS1* construct into embryogenic calli of sweet potato (cultivar Xu 29; Fig. 5a). Three regenerated transgenic lines (L11, L17, and L19) with the highest *IbPSS1* transcript levels were used for further physiological characterization (Fig. 5b). NaCl (200 mM) was administered to uniformly rooted wild-type (WT) and *IbPSS1*-transgenic seedlings for 9 days. The results showed that, compared with the WT, the *IbPSS1*-transgenic lines exhibited obviously enhanced salt tolerance (Fig. 5c). Compared with the WT, the salinized transgenic lines had a markedly higher survival rate (Fig. 5d). Consistent with the salt-tolerant phenotype, the elongation zone of the salinized roots of the three transgenic lines had a significantly lower Na^+^ accumulation level than did that of the WT (Fig. 5e, f). In addition, the elongation and mature zones of the salinized roots of the three transgenic lines had significantly higher net Na^+^ efflux than did those of the WT (Fig. 5g). Taken together, these findings show that *IbPSS1* overexpression improves salt tolerance at the whole-plant level by accelerating Na^+^ exclusion in the roots. Moreover, the PM Ca^2+^-permeable channel sensitivity to NaCl and H_2_O_2_ was enhanced in the roots of the *IbPSS1*-overexpressing seedlings (Fig. 5h, j).

**Fig. 6:**
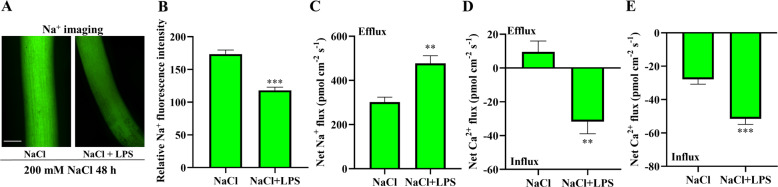
Exogenous application of lysophosphatidylserine (LPS) decreases cellular Na^+^ accumulation by stimulating Na^+^ extrusion and Ca^2+^ influx in sweet potato roots. Sweet potato (WT, Xu 29) roots were pretreated with 50 μM LPS for 24 h and then subjected to NaCl stress (200 mM) for another 48 h. **a** Effects of LPS on cellular Na^+^ accumulation. Na^+^ accumulation in the root elongation zone of LPS-pretreated or nonpretreated seedlings. A typical image (of 22) is shown. Bar = 0.3 mm. **b** Quantification of Na^+^ fluorescence intensity. **c** Effects of LPS on the steady-state flux of Na^+^. The Na^+^ flux was measured from the root elongation zone after 48 h of NaCl stress. *n* = 16 from six individual seedlings. The SEs are represented by the bars. **d** Effects of LPS on NaCl-induced Ca^2+^ flux. The Ca^2+^ flux was measured from the root elongation zone (1.5, 2.5, and 3 mm from the tip) after 10 min of NaCl treatment. *n* = 12 from six individual seedlings. **e** Effects of LPS on H_2_O_2_-induced Ca^2+^ flux. The Ca^2+^ flux was measured from the root elongation zone (1.5, 2.5, and 3 mm from the tip) after the addition of 10 mM H_2_O_2_. *n* = 12 from six individual seedlings. For all the histograms, the columns labeled with “*,” “**,” and “***” indicate significant differences at *P* < 0.05, *P* < 0.01, and *P* < 0.001, respectively.

Exogenous application of lysophosphatidylserine (LPS) restores the PS content in and rescues the growth defects of *AtPSS1* Arabidopsis mutants^17^. The effects of exogenous LPS on Na^+^ accumulation and Na^+^ flux after salinity stress were evaluated for their association with the *IbPSS1*-modulated cellular processes involving PS. The Na^+^ fluorescence in the root elongation zone of the LPS-pretreated seedlings (Xu 29, WT) was obviously lower than that in the nontreated Xu 29 seedlings after 48 h of NaCl stress (200 mM) (Fig. 6a, b). These findings suggest that the Na^+^ accumulation decreased. Consistent with Na^+^ accumulation, Na^+^ efflux increased by 65% in the root elongation zone of the LPS-pretreated Xu 29 after 48 h of NaCl stress compared with that in the nontreated roots (Fig. 6c). Moreover, the mean Ca^2+^ influx rate triggered by NaCl stress (150 mM; the Ca^2+^ flux measurement was started after 10 min of NaCl treatment; Fig. 6d) and H_2_O_2_ (10 mM; the Ca^2+^ flux measurement was started immediately after H_2_O_2_ treatment; Fig. 6e) was reinforced more in the LPS-pretreated roots than in the nontreated roots. These results suggest that PS modulates PM Ca^2+^-permeable channel sensitivity to NaCl and H_2_O_2_.

## Discussion

Although the *A. tumefaciens*-mediated transformation method has been used for many sweet potato genotypes and although various genes have been characterized in detail^30^, current embryogenic callus-based transformation procedures of sweet potato still limit functional genomics in this important food crop species because they are time consuming and laborious. In the present study, a convenient and highly efficient method in which *A. rhizogenes* is used to induce the formation of TRs in intact sweet potato cuttings was established (Fig. 2a). This root transgenic system is similar to a previously reported strategy in woody plants^26,31^. Given that sweet potato is vegetatively propagated, an in vivo root transgenic system is not required for the sterilization of explants prior to *A. rhizogenes* infection^26,31^. The fluorescent marker DsRed used here helps to identify TRs from nontransgenic ARs of the same seedling^31^ (Fig. 2b). The nonoverlapping emission spectra between DsRed and green fluorescence-based physiological analyses enable the widespread application of this system through fluorescence microscopy or confocal microscopy, such as imaging Na^+^ accumulation by the use of CoroNa^TM^ Green (this study; Figs. 2d, 5e, and 6a) or recoding cytosolic Ca^2+^ dynamics by the use of Ca^2+^ fluorescent probes and genetically encoded Ca^2+^ sensors (GCaMP3 and GCaMP6)^32,33^.

The cellular functions of genes of interest in different biological backgrounds could be determined within several days by combining this transgenic method with other physiological and molecular analyses (Fig. 2a). This strategy facilitates the determination of the biological function of genes of interest and accelerates the application of these genes to the genetic improvement of sweet potato through CRISPR-based technology. For example, by using the combination of NMT with this root transgenic system within 40 days, we revealed the novel role of *IbPSS1* in stimulating PM Na^+^/H^+^ antiport activity in salinized sweet potato roots (Figs. 2 and 3). Importantly, the transgenic hairy roots induced by *A. rhizogenes* were anatomically similar to the primary roots and the ARs^34^. In addition, in the present study, the cellular processes influenced by the gene of interest (*IbPSS1*) did not differ between the *A. rhizogenes*- and *A. tumefaciens*-mediated transgenic systems (Figs. 2–5). Thus, our root transgenic system serves as a fast, convenient, and reliable tool for functional genomics in sweet potato. Given that each single TR represents an independent transformation event, this system can be a useful tool for testing the gene-editing efficiency among different CRISPR-Cas9/Cas12a/Cas12b variants or for developing regenerated and genome-edited germplasms of this autohexaploid crop species^34–36^.

PS plays an indispensable role in regulating plant development. AtPSS1-mediated alterations in PS contents in the PM are important regulators of small GTPase signaling during Arabidopsis root development^24^. Genetic disruption of PS biosynthesis decreases heterozygote fertility by inhibiting pollen maturation in Arabidopsis^19^. In the present study, we found that homologously overexpressed *IbPSS1* facilitates Na^+^ efflux and H^+^ influx in salinized sweet potato roots (Fig. 3), indicating the enhancement of PM Na^+^/H^+^ antiporter activity^27–29^. This enhanced Na^+^/H^+^ antiport capability promotes Na^+^ homeostasis at the cellular level and increases salt tolerance at the whole-plant level (Fig. 5). Evidence from in vitro experiments showed that PS activates PM H^+^-ATPase activity in isolated PM vesicles of rice culture cells^37^. Moreover, our previous work showed that the exogenous addition of PS to sweet potato mesophyll cells stimulates H^+^ extrusion and PM H^+^-ATPase activity under saline conditions^25^. The potentially enhanced PM H^+^-ATPase activity, which generates more electrochemical H^+^ gradients to promote PM Na^+^/H^+^ antiporters^1,2^, may contribute to the enhanced Na^+^ extrusion and Na^+^ homeostasis in *IbPSS1*-overexpressing roots.

Salt stress increases [Ca^2+^]_cyt_ concentration by activating PM Ca^2+^-permeable channels, and the expulsion of excess intracellular Na^+^ involves the Ca^2+^-related SOS signaling pathway^2,16^. H_2_O_2_, which is triggered by high-salinity stress, facilitates the activation of PM Ca^2+^-permeable channels and the formation of a specific [Ca^2+^]_cyt_ signature, thereby contributing to the increase in PM Na^+^/H^+^ antiporter activity^3–6^. We revealed that the salt- and H_2_O_2_-induced Ca^2+^ flux patterns were altered in the *IbPSS1*-overexpressing roots or exogenous LPS-pretreated roots (Figs. 4, 5h–5j, 6d, e), suggesting that PM Ca^2+^-permeable channels are targets of PS under stressful conditions. *IbSOS1* transcript levels exhibited no difference between the *IbPSS1*-overexpressing roots and the ARs (Fig. S4). Thus, the increased influx of Ca^2+^ across the PM possibly contributed to the activation of IbSOS1 at the posttranslational level via the SOS pathway, in which the phosphorylation of IbSOS1 may be enhanced by the reinforced Ca^2+^ signaling^2^. Given that H_2_O_2_ is an important signaling molecule involved in the activation of PM Ca^2+^-permeable channels in plants under various environmental stresses^38^, we hypothesize that the PSS-mediated variations in PS abundance in the PM play multiple signaling roles in the plant response to environmental changes. Although we did not observe salt-induced transcriptional changes in *IbPSS1* in sweet potato roots (Fig. 2c), high-throughput lipidomic profiling has proven that the PS abundance in different plant tissues changes in response to various environmental stresses^25,39,40^.

In plant cells, PS is necessary and sufficient for establishing and maintaining the PM electrostatic signature, which may contribute to the specific targeting of proteins to the PM within the electrostatic region^17^. *IbPSS1* overexpression may alter the abundance of PS within cells and its distribution in the PM, thereby shifting the electrostatic gradient and the targeting pattern of specific proteins in sweet potato root cells in response to salt and H_2_O_2_ stimuli^17^. Annexins are soluble proteins with multiple functions in plants^41^. Several lines of evidence suggest that annexins mediate the PS modulation of cellular responses to salt and H_2_O_2_. (1) Like vertebrate annexins, plant annexins contain a conserved PS binding site^41^. (2) Plant annexins (e.g., AtANN1 and AtANN4 from Arabidopsis) function as Ca^2+^-permeable channels (or cofactors of channels) that fine tune Ca^2+^ influx across the PM and the increase in [Ca^2+^]_cyt_ in response to NaCl and H_2_O_2_ stimuli^42–44^. (3) Evidence from proteomic analyses of different cell fractions shows that NaCl stress stimulates the relocation of AtANN1 from the cytosol to root cell membranes^45^. (4) The ectopic expression of *GhANN8b*, which encodes an annexin from cotton (*Gossypium* spp.), stimulates salt-induced Ca^2+^ influx and Na^+^/H^+^ antiport activity across the PM in Arabidopsis roots^46^. Considering these lines of evidence and our findings, we suggest that annexins are the most probable targets downstream of IbPSS1-mediated PS synthesis and are involved in the regulation of Ca^2+^ influx and Na^+^/H^+^ antiporter capacity across the PM under saline conditions.

Na^+^ in the soil may be sensed extracellularly, intercellularly, or by transporters or channels at the PM in plant roots^1^. In Arabidopsis, external Na^+^ binds to GIPC sphingolipids outside of the PM and activates PM Ca^2+^-permeable channels to induce downstream responses to salinity, including an increase in [Ca^2+^]_cyt_ and the activation of PM Na^+^/H^+^ antiporters via the SOS pathway^16^. Thus, GIPC sphingolipids have been identified as cell surface Na^+^ receptors required for tolerance to salinity in Arabidopsis^16^. Different Na^+^ sensing routes may exist and may be responsible for the initiation of multiple salt defensive responses in plants^1^. Considering that Na^+^ binds specifically to PS vesicles and that PS mainly localizes in the cytosolic leaflets of the PM^17,24,47^, PS may function as an intracellular Na^+^ sensor to modulate downstream responses to salinity, including annexin-mediated Ca^2+^ influx into the cell, the formation of a specific [Ca^2+^]_cyt_ signature, the regulation of PM Na^+^/H^+^ antiporter activity, and PM H^+^-ATPase activity via a specific Ca^2+^-activated molecular pathway^48^. However, this hypothesis still needs further confirmation.

In conclusion, this study provides a fast, convenient, and reliable transgenic method for functional genomic studies of sweet potato. Our results showed that IbPSS1-mediated PS production is involved in the mediation of salt- and H_2_O_2_-induced Ca^2+^ transport across the PM and revealed that IbPSS1 positively regulates PM Na^+^/H^+^ antiporter activity and cellular Na^+^ homeostasis in the roots under saline conditions, thereby contributing to the enhancement of salt tolerance at the whole-plant level in sweet potato. To the best of our knowledge, this is the first study to investigate the influence of PSS on Na^+^ homeostasis and salt tolerance in plants. This study highlights the signaling role of PSS-mediated PS synthesis in mediating Na^+^ sensing and downstream defensive responses. However, further studies using antisense techniques are still needed to determine whether basic PS synthesis is required for the activation of PM Ca^2+^-permeable channels and PM Na^+^/H^+^ antiporters under saline conditions.

## Materials and methods

### Plant materials and growth conditions

The sweet potato varieties utilized in this study were obtained from the Xuzhou Institute of Agricultural Sciences in Jiangsu Xuhuai district, Xuzhou, Jiangsu, China. For stem cutting propagation, sweet potato seedlings were cultivated in pots (peat moss:loamy soil =1:1) placed inside a clean greenhouse whose temperature was 25 °C and whose photoperiod was 16 h. After enough seedlings were obtained, the stems were removed and used to induce TRs through *A. rhizogenes*.

### Isolation of IbPSS1 and vector construction

Young leaves of Xu 29 were used to extract total RNA by using a TRIzol Reagent Kit. Total RNA was purified, and first-strand cDNA was synthesized by using HiScript II Q Select RT SuperMix (Vazyme). The conserved cloning primer (forward: 5′-ATGGAGCCTAATGGTCATAGAAGGAGTA-3′; reverse: 5′-TCATTGCCGCTTTCTCTTCATCAATTGC-3′) for *IbPSS1* was designed according to the homologous gene from *Ipomoea triloba* (GenBank ID: XM_031245646.1), which is a wild relative of cultivated sweet potato. The cDNA was amplified by PCR using StarMAX DNA Polymerase (Takara, Japan), and the PCR product was subsequently purified and cloned into a pGEM-T vector for sequencing and alignment. *IbSOS1* was also isolated from the sweet potato cultivar Xushu 22 (cloning primer: forward-5′-ATGACTTCCATGCTGGTGACG-3′; reverse-5′-CTAGCGAAAAGACAGTGTGCTTGG-3′).

The coding region of *IbPSS1* or *IbSOS1* and *DsRed* was inserted into a pCAMBIA0390 expression vector. Three constructs (*pCaMV35S::DsRed*, *pUBI.U4::IbSOS1-CaMV35S::DsRed*, and *pUBI.U4::IbPSS1-CaMV35S::DsRed*) were used for *A. rhizogenes-*mediated transformation. The coding region of *IbPSS1* was inserted into a pCAMBIA1380 expression vector for *A. tumefaciens-*mediated transformation. Furthermore, two transient expression vectors expressing *IbPSS1* and the PS sensor C2^LACT^-GFP were constructed using pRI201-AN as the backbone vector (*pUBI.U4::IbPSS1* and *pCaMV35S::C2*^*LACT*^*-GFP*).

### Phylogenetic analysis

The full-length amino acid sequences of IbPSS1 and its orthologs in other species were obtained from the UniProt and Phytozome databases. The amino acid sequences of the PSS protein were aligned by ClustalW, with the default settings. The conserved sequences were shaded at four levels by GeneDoc. A neighbor-joining phylogenetic tree of PSS proteins was constructed using the MEGA program (version 7.0.14), with 1000 bootstrap replications. The accession numbers of genes utilized in the present study are listed in Table S1.

### PS abundance assay using the genetically encoded fluorescent sensor C2^LACT^-GFP

The *pUBI.U4::IbPSS1* and *pCaMV35S::C2*^*LACT*^*-GFP* vectors were introduced into *A. tumefaciens* strain GV3101 by using the freeze–thaw method. *A. tumefaciens* was then resuspended in infiltration buffer (10 mM MES-KOH, 10 mM MgCl_2_, 200 μM acetosyringone (AS); pH 5.6) and diluted to an OD_600_ of 0.5. Equal volumes of *A. tumefaciens* solution containing the two constructs were mixed, and 1 mL of the mixture was infiltrated into the leaves of 4-week-old *N. benthamiana* plants by using a needleless syringe. EV instead of the *pUBI.U4::IbPSS1* construct served as a control. The fluorescence image was visualized 4 days post-inoculation under an Olympus BX63 epifluorescence microscope using the same settings. Image-Pro Plus 6.0 software was then used to quantify the relative fluorescence intensity in the PM region.

### A. rhizogenes-mediated transformation

Uniform sweet potato (Xu 29 and Zi 8) cuttings were collected for agro-infiltration using the following procedure. The three constructs were introduced into *A. rhizogenes* strain K599 and cultured in 5 mL of YPD liquid media consisting of 20 mg/L rifampicin and 50 mg/L kanamycin under shaking conditions (200 rpm) at 28 °C for 12–14 h. After the OD_600_ value reached 0.5, the cultures were centrifuged at 5000 rpm for 10 min at room temperature and then resuspended in infiltration buffer (10 mM MES-KOH, 10 mM MgCl_2_, 100 μM AS; pH 5.2). A 1.5 mL aliquot of the *A. rhizogenes* suspension was then injected into the stem of sweet potato cuttings by using a syringe with a needle. Afterward, the cuttings were transplanted into the soil and grown in a high-humidity environment. The transgenic positive plants were collected for further analysis after 3 weeks of culture in the greenhouse. The induction rate of the TRs was calculated as follows: (number of plants with TRs/total number of injected plants) ×100.

### Generation of transgenic sweet potato overexpressing IbPSS1 via A. tumefaciens-mediated transformation

The *pCaMV35S::IbPSS1* construct was introduced into *A. tumefaciens* EHA105, cultured overnight on a shaker (200 rpm, 28 °C) in YPD liquid media consisting of 12.5 mg/L hygromycin and 200 μM AS, and then transformed into embryogenic calli of sweet potato (Xu 29) using a reported previously *A. tumefaciens*-based method^49^. Transgenic calli were screened in media containing an appropriate concentration of hygromycin. Transgenic positive lines were verified by genomic DNA PCR analysis. qRT-PCR analysis was then performed to choose *IbPSS1*-overexpressing transgenic lines for further physiological analysis (*IbPSS1* qRT-PCR primers: forward, 5′-CACCACCGTAGGATCTTTCAGG-3′; reverse, 5′-GTGATGATGCTCGCCAGTTTAT-3′). Three transgenic lines were selected and cultivated in the greenhouse for stem cutting propagation.

### Salt treatment

#### Transgenic roots

Sweet potato seedlings with TRs (which fluoresced red under the excitation of a portable fluorescent lamp) were selected and immersed in hydroponic solution (1/4-strength Hoagland solution) for 24 h. Afterward, NaCl stress was started by adding the required amount of NaCl (final concentration of 200 mM) to the hydroponic solution. Before or after 24 h of NaCl stress, the ARs and TRs were collected to measure gene expression, intracellular Na^+^ accumulation, steady-state Na^+^ and H^+^ fluxes, and transient Ca^2+^ flux kinetics.

#### Transgenic plants

After obtaining enough seedlings, the shoots (with 4–5 mature leaves) of the WT and three transgenic lines were cut and cultivated in hydroponic solution to initiate AR growth for 10 days. Afterward, uniformly rooted seedlings were subjected to NaCl stress (200 mM) for another 9 days. The phenotype and survival rate were recorded at corresponding time points, after which the roots were collected to measure the intracellular Na^+^ accumulation, steady-state Na^+^ flux, and transient Ca^2+^ flux.

#### Exogenous LPS treatment

Rooted WT (Xu 29) seedlings were immersed in hydroponic solution in the absence or presence of 50 μM LPS (Avanti Polar Lipids, 850092 P) for 24 h. The required amount of NaCl (200 mM) was then added. The fine roots were collected to measure intracellular Na^+^ accumulation and Na^+^ flux after 48 h of NaCl treatment. The Ca^2+^ flux response to NaCl and H_2_O_2_ was also measured after 10 min of treatment.

### Visualization of Na^+^ in root cells

CoroNa^TM^ Green AM (Life Technologies), which is a Na^+^-specific fluorescent dye, was used to detect the accumulation of Na^+^ in sweet potato root cells^5^. The roots were collected after NaCl treatment and placed in a centrifuge tube containing fresh incubation solution (200 mM NaCl, 20 μM CoroNa^TM^ Green AM (Life Technologies), and 0.02% pluronic acid) for 2 h. Afterward, they were washed several times with distilled water. An Olympus BX63 epifluorescence microscope was subsequently used to visualize intracellular Na^+^ accumulation (as indicated by the green fluorescence). All images were taken using the same settings and exposure times to allow direct comparisons. Image-Pro Plus 6.0 software was used to quantify the relative fluorescence intensity in the different root zones.

### Analysis of transcript levels

Total RNA was extracted from the roots using TRIzol reagent (Takara Bio Inc., Japan). qRT-PCR was conducted as described previously^50^. Primers were designed to target *IbSOS1* (forward: 5′-GGACTCCTCAGTGCTACT-3′; reverse: 5′-CATCGTTCATCAAGGATTCTC-3′) and *IbPSS1* (forward: 5′-CACCACCGTAGGATCTTTCAGG-3′; reverse: 5′- GTGATGATGCTCGCCAGTTTAT-3′). The relative gene expression level was normalized to the reference gene (*IbGAPDH*; forward, 5′-ATACTGTGCACGGACAATGG-3′; reverse, 5′-TCAGCCCATGGAATCTCTTC-3′). The 2^−∆∆Ct^ method was then used to calculate the relative expression levels.

### Measurement of ion fluxes

The net ion fluxes were measured using the NMT system^5,25,27,50^. Ion-selective microelectrodes were constructed in accordance with our previously reported procedures^27^. The ion-selective microelectrodes were calibrated before recording the flux: (1) for Na^+^, 0.1, 0.5, and 1.0 mM NaCl (pH = 5.7) were used; (2) for H^+^, solutions with a pH of 5.0, 6.0, and 7.0 were used; and (3) for Ca^2+^ and K^+^, 0.1, 0.5 and 1.0 mM CaCl_2_ or KCl (150 mM NaCl or 10 mM H_2_O_2_ was added to the background solution; pH=5.7) was used. Microelectrodes with Nernstian slopes >52 mV per decade for Na^+^, H^+^, and K^+^ (26 mV per decade for Ca^2+^) were used. The ion flux was calculated in accordance with previously described methods^5,25,27,50^.

### Steady-state Na^+^, H^+^ and Ca^2+^ flux measurements

Fine roots with 3–4 cm apices were collected from NaCl-stressed ARs and TRs and from WT and *IbPSS1* transgenic lines. For Na^+^ and H^+^ flux recording, the roots were washed several times with the measuring solution (0.5 mM KCl, 0.1 mM MgCl_2_, 0.1 mM NaCl and 0.1 mM CaCl_2_; pH=5.7), followed by a 30 min equilibration in the measuring solution^5,27^. These root segments were then immobilized in the bottom of the measuring chamber that contained 10 mL of fresh measuring solution. Ion fluxes were recorded along the root axis: in the meristem zone (300, 400, and 500 μm from the tip, only for the *IbSOS1* experiments), elongation zone (1.5, 2.0, and 2.5 mm from the tip), and mature zone (10, 12, and 15 mm from the tip). Recording was continuously conducted for 3–5 min at each measuring point in the three root zones.

For exogenous LPS experiments, the salt-induced changes in Na^+^ flux or the salt- and H_2_O_2-_induced alterations in Ca^2+^ flux in the root elongation zone were measured after 48 h or after 10 min of the corresponding treatments. The same measuring solution was used in this series of experiments.

### Transient Ca^2+^ and K^+^ flux measurements

Fine roots with 3–4 cm apices were collected from ARs and TRs and from WT and *IbPSS1* transgenic lines to measure the transient Ca^2+^ flux. The roots were fixed in the measuring solution (0.5 mM KCl, 0.1 mM MgCl_2_, 0.1 mM NaCl and 0.1 mM CaCl_2_; pH=5.7) for 30 min. The Ca^2+^ flux was recorded from the root elongation zone (2 mm from the tip) for 5 min before salt and H_2_O_2_ treatment. Afterward, the salt and H_2_O_2_ treatments were applied by adding NaCl (prepared with measuring solution, with a final concentration of 150 mM) and H_2_O_2_ (prepared with measuring solution, with a final concentration of 10 mM) to the measuring solution. The transient ion fluxes in the root elongation zone were measured for another 20 min. The NaCl-induced transient K^+^ flux kinetics in the ARs and TRs were also measured as described above.

### Statistical analysis

The data were analyzed using ANOVA. “*”, “**”, and “***” indicate significant differences at *P* < 0.05, *P* < 0.01, and *P* < 0.001, respectively.

## Supplementary information

Supplementary Table S1

Supplementary Figure S1

Supplementary Figure S2

Supplementary Figure S3

Supplementary Figure S4

Supplementary Figure S5
