# Supplementary material for: Overexpression of phosphatidylserine synthase IbPSS1 affords cellular Na+ homeostasis and salt tolerance by activating plasma membrane Na+/H+ antiport activity in sweet potato roots
Source: Hortic Res. 2020 Aug 1;7:131. doi: 10.1038/s41438-020-00358-1 (PMC7395154; doi:10.1038/s41438-020-00358-1)
Supplement: Supplementary file 1 — Supplementary Table S1 [file 41438_2020_358_MOESM1_ESM.docx]

Table S1: Accession number of genes used in phylogenetic analysis.

| Gene_symbol | Database | Gene accession number |
| --- | --- | --- |
| PtPSS1;1 | Phytozome | Potri.010G117500 |
| PtPSS1;2 | Phytozome | Potri.008G126100 |
| VvPSS1 | Phytozome | GSVIVT01011808001 |
| GmPSS1;1 | Phytozome | Glyma.10g268300 |
| GmPSS1;2 | Phytozome | Glyma.20g122800 |
| OsPSS1 | Phytozome | LOC_Os01g02890 |
| ZmPSS1;1 | Phytozome | GRMZM2G110834 |
| ZmPSS1;2 | Phytozome | GRMZM2G095757 |
| SbPSS1 | Phytozome | Sobic.003G095800 |
| AtPSS1 | Phytozome | AT1G15110 |
| SlPSS1;1 | Phytozome | Solyc04g007520 |
| SlPSS1;2 | Phytozome | Solyc05g008580 |
| HsPSS1 | UniProt | P48651 |
| HsPSS2 | UniProt | Q9BVG9 |
| CgPSS1 | UniProt | Q00576 |
| CgPSS2 | UniProt | O08888 |
