# Supplementary figures and images for: Overexpression of phosphatidylserine synthase IbPSS1 affords cellular Na+ homeostasis and salt tolerance by activating plasma membrane Na+/H+ antiport activity in sweet potato roots
Source: Hortic Res. 2020 Aug 1;7:131. doi: 10.1038/s41438-020-00358-1 (PMC7395154; doi:10.1038/s41438-020-00358-1)

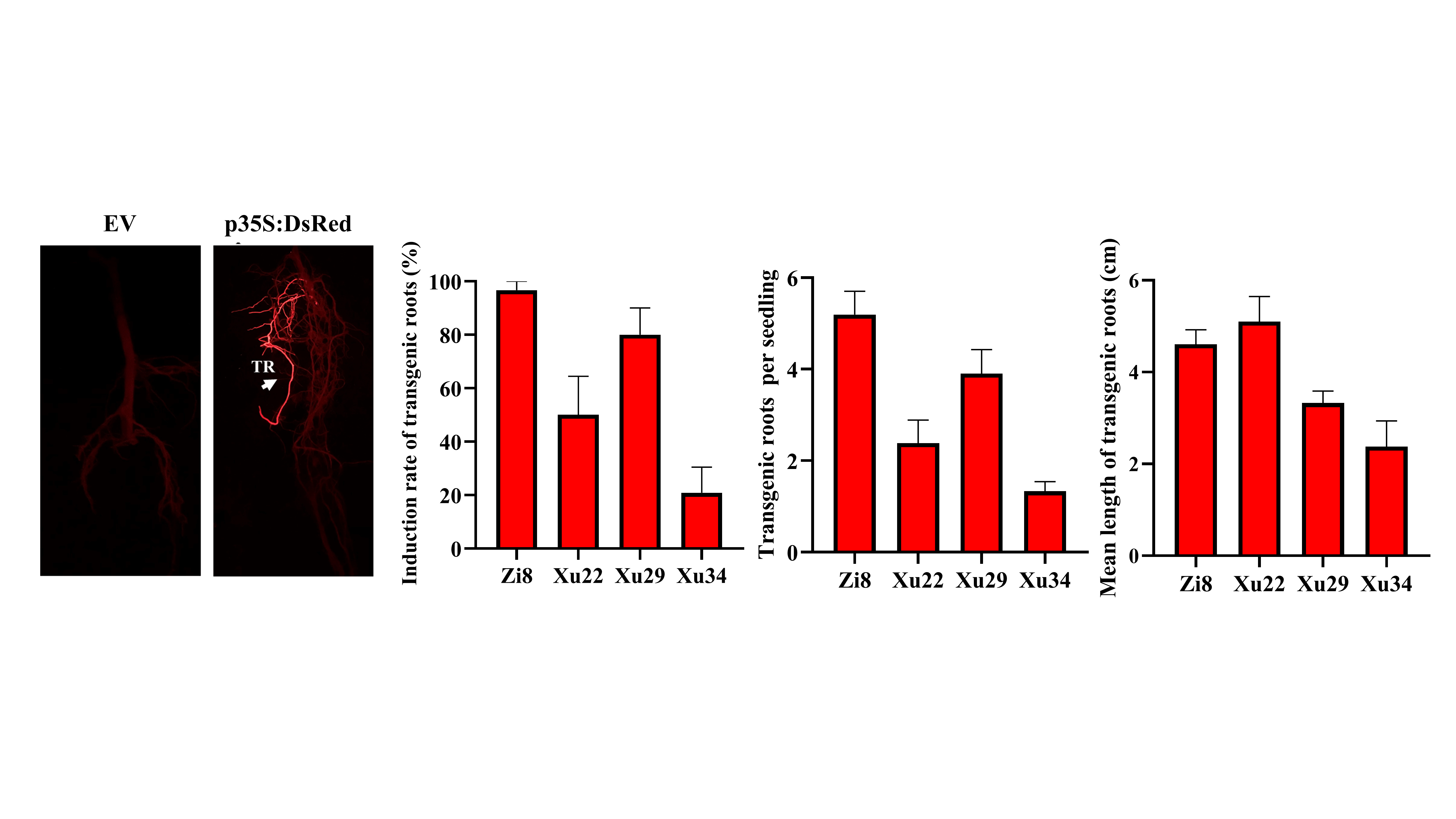

Supplement: Supplementary file 2 — Supplementary Figure S1 [file 41438_2020_358_MOESM2_ESM.tif]

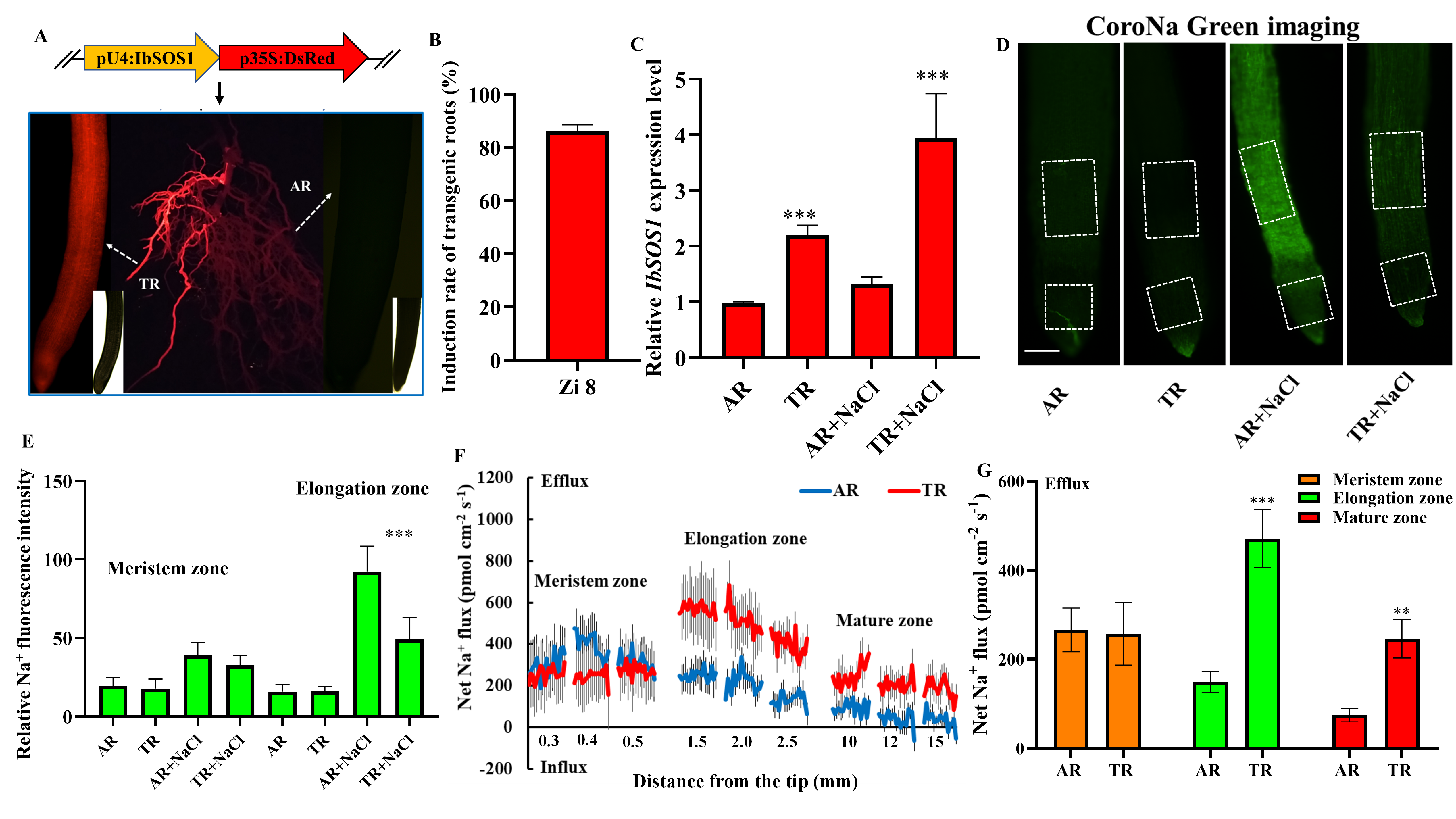

Supplement: Supplementary file 3 — Supplementary Figure S2 [file 41438_2020_358_MOESM3_ESM.tif]

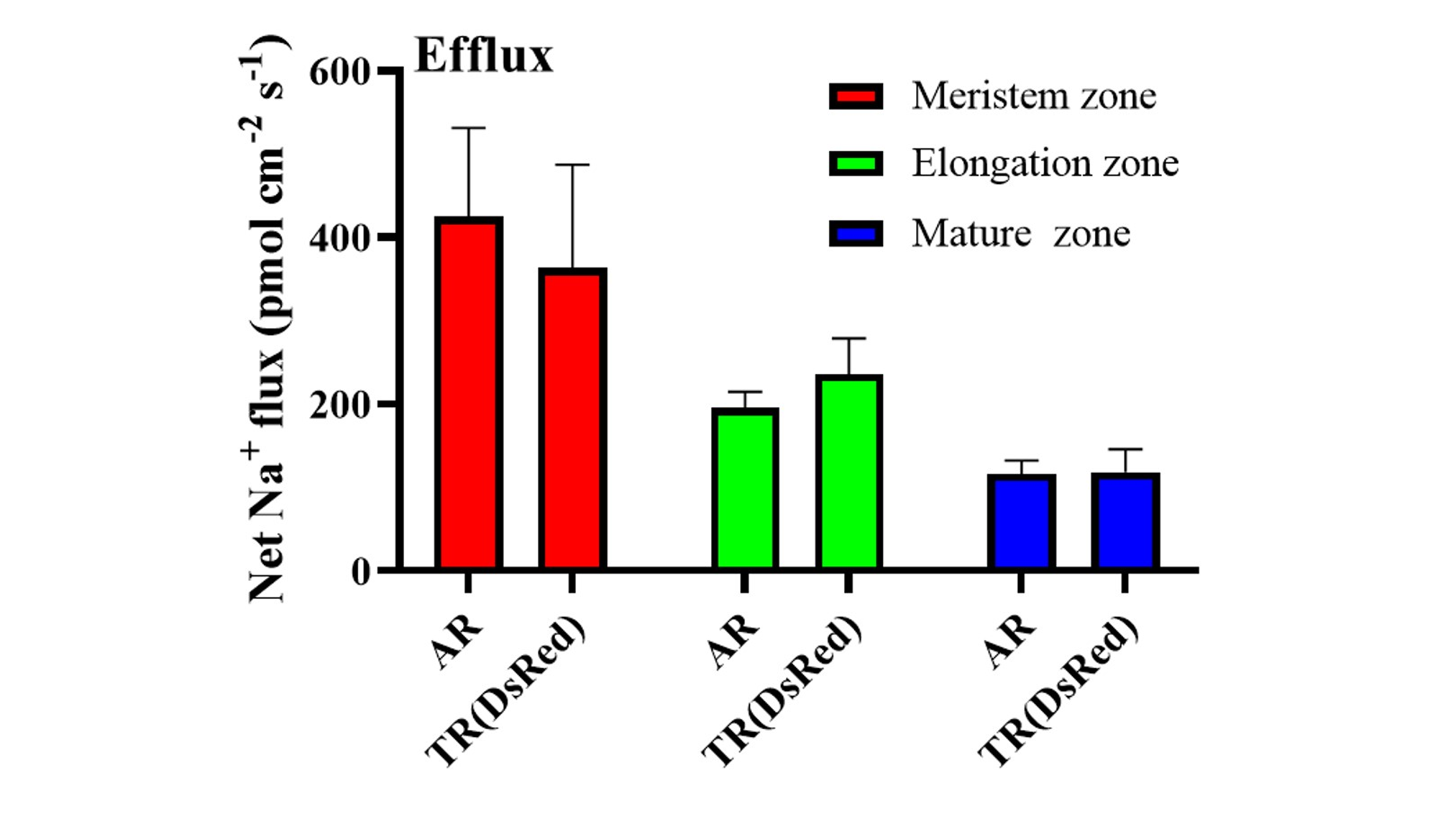

Supplement: Supplementary file 4 — Supplementary Figure S3 [file 41438_2020_358_MOESM4_ESM.tif]

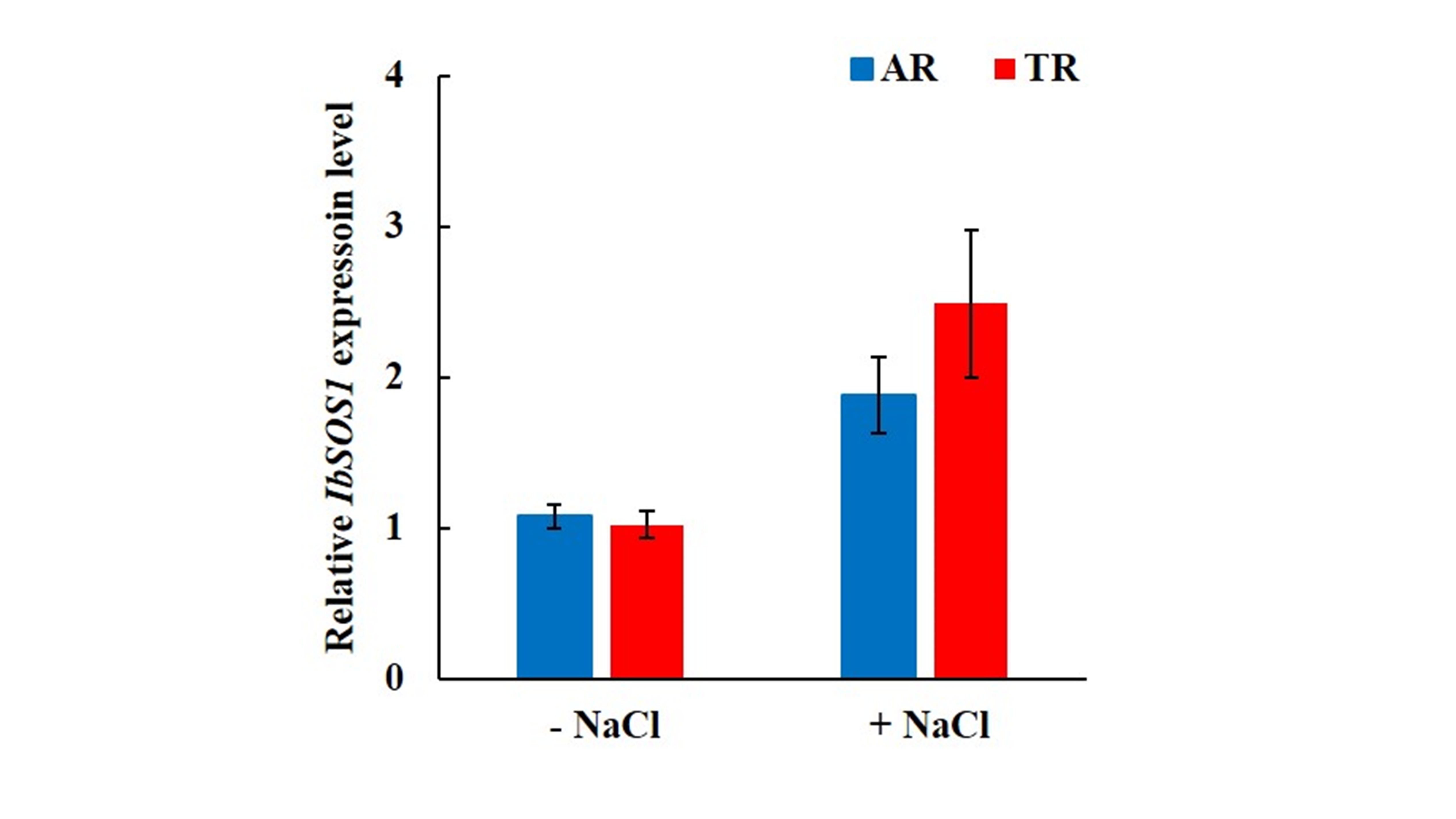

Supplement: Supplementary file 5 — Supplementary Figure S4 [file 41438_2020_358_MOESM5_ESM.tif]

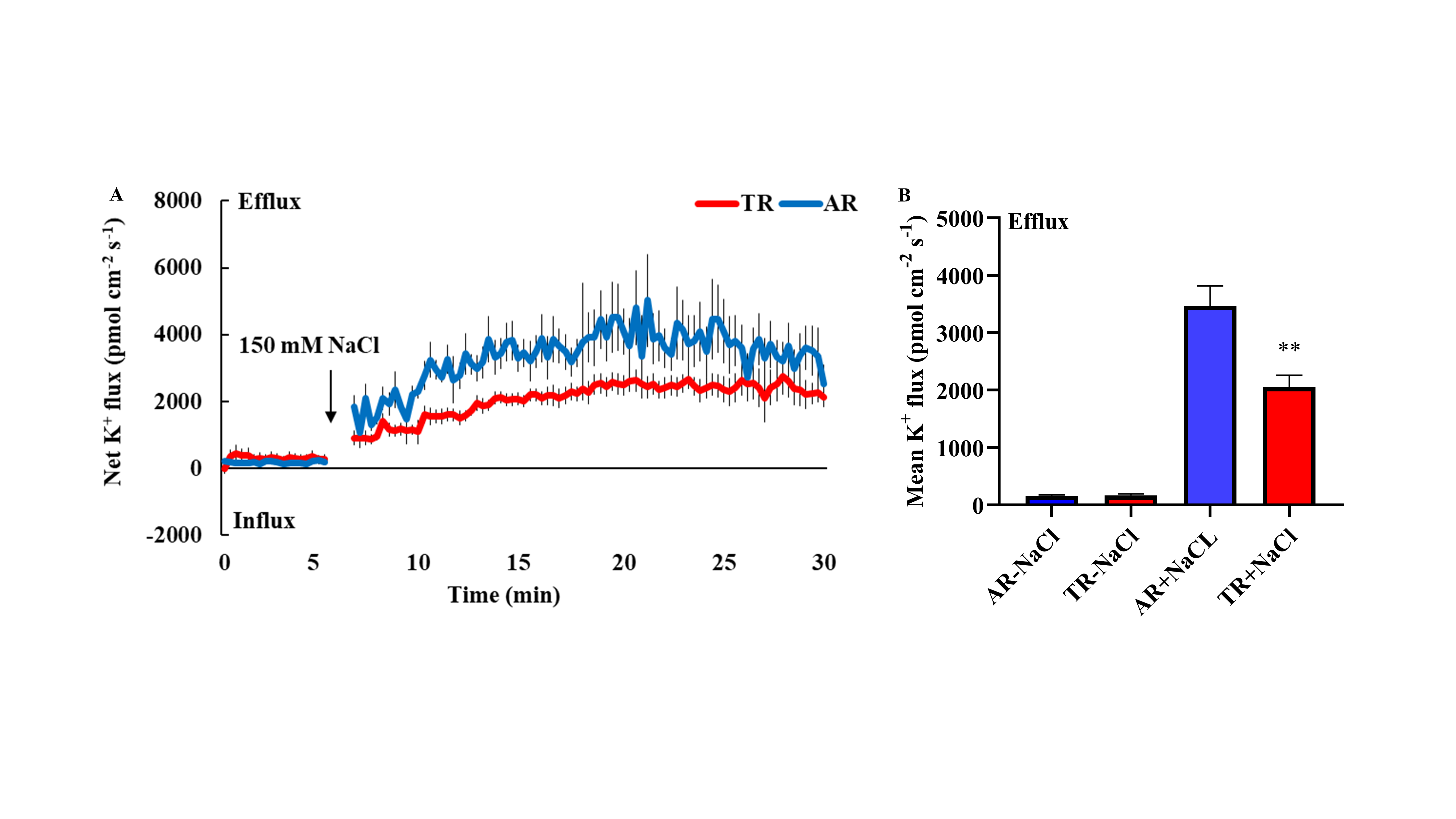

Supplement: Supplementary file 6 — Supplementary Figure S5 [file 41438_2020_358_MOESM6_ESM.tif]
